# Efficacy, safety and recurrence of new progestins and selective progesterone receptor modulator for the treatment of endometriosis: a comparison study in mice

**DOI:** 10.1186/s12958-018-0347-9

**Published:** 2018-04-03

**Authors:** Bo Liang, Ling Wu, Hui Xu, Chun Wai Cheung, Wen Ying Fung, Sze Wai Wong, Chi Chiu Wang

**Affiliations:** 1 Department of Obstetrics and Gynaecology, The Chinese University of Hong Kong, c/o 1st Floor, Special Block E, Prince of Wales Hospital, Shatin, Hong Kong; 2 Reproduction and Development Laboratory, Li Ka Shing Institute of Health Sciences, The Chinese University of Hong Kong, Shatin, Hong Kong; 3 School of Biomedical Sciences, The Chinese University of Hong Kong, Shatin, Hong Kong

**Keywords:** Progestin, Selective progesterone receptor modulator, Endometrium

## Abstract

**Background:**

Current medical treatments for endometriosis are very limited. Progestin and selective progesterone receptor modulators (SPRM) are developed but their efficacy, safety, mechanism and recurrence in endometriosis are not fully studied.

**Methods:**

In order to compare therapeutic, side effects and therapeutic actions of Esmya, Duphaston and Dienogest in endometriosis. Experimental endometriosis was induced by either intraperitoneal or subcutaneous mouse endometrium transplantation. Lesion size, weight and histology at the end of intervention were compared. Expression of related markers in the endometriotic lesions were examined. Body, uterus and ovary weights, endometrial glands and thickness (ETI), and follicle count were measured. For recurrent study, lesion growth before and after intervention was monitored.

**Results:**

After Esmya, Duphaston, Dienogest treatment, lesion size and weight were significantly decreased. Proliferation Pcna expression was significantly decreased in all groups, but proliferation cells were significantly decreased only in Duphaston group. Apoptosis Mapk1 expression and TUNEL-positive cells were significantly increased in Duphaston group. Adhesion Mmp2 and Itgavβ3 expression were significantly increased in Esmya group. Plau, Hif1α and Vegfa expression, peritoneal fluid PGE2 levels, and ERα and ERβ expression were not affected; while PR expression was significantly lower in all groups. Endometrial gland count in uterus was significantly increased in Dienogest group, ETI was significantly decreased in Duphaston group, and AFC were significantly increased in Esmya group. Upon treatment cessation, lesion growth rebound quickly in Dienogest and Duphaston groups, but slowly in Esmya group.

**Conclusion:**

Esmya, Duphaston and Dienogest are effective anti-endometriosis drugs targeting proliferation, apoptosis and adhesion. Esmya, Duphaston and Dienogest are all well tolerable, although endometrial glandular hyperplasia was found in Dienogest, endometrial atrophy in Duphaston, follicle accumulation in Esmya.

**Electronic supplementary material:**

The online version of this article (10.1186/s12958-018-0347-9) contains supplementary material, which is available to authorized users.

## Background

Endometriosis is a common and chronic benign gynecological disorder characterized by the presence of endometrial-like tissues outside the uterine cavity. It affects around 10% of reproductive women, and up to 24–50% of infertile women [1, 2]. Although endometriosis is an estrogen-dependent disease, etiological studies showed that pathogenesis of endometriosis is complex and multifactorial [3–6]. The most widely accepted and scientifically supported theory for the occurrence of endometriosis is retrograde menstruation since the anatomical locations and histochemical characteristics of endometriotic lesions [4, 7]. Clinical symptoms of endometriosis include but not limited to pelvic pain, dysmenorrhea, dyspareunia and infertility [8], which have significant impacts on both general and mental health of afflicted individuals [9, 10]. Current available treatment of endometriosis, including removal surgery and hormonal medication [11], are still not ideal for reproductive women who would like to preserve their fecundity without any side-effect and recurrence.

Seeking targeted therapy with minimal side-effect has been going on for several decades, but there is still no better medication. Recently selective progesterone-receptor modulators (SPRM) and new generation progestins have been speculated or indicated as potential treatment for endometriosis [12–14]. For example, Ulipristal acetate (Esmya) is a SPRM, which has been used for uterine fibroids for a while [15]. Esmya can reduce the volume of uterine fibroid by inhibiting proliferation and increasing apoptosis [16, 17]. It may also work on endometriosis, but its therapeutic effects on endometriosis are still not fully evaluated. Duphaston contains dydrogesterone, a progestin derived from 9β,10α-progesterone [18], which can relieve pain in endometriosis and does not interrupt pregnancy during treatment. There is no conclusive evidence to support its therapeutic use in endometriosis [13, 19]. Dienogest is another progestin derived from 19- nortestosterone [18], which have been used to relief symptoms of endometriosis [20]. It can inhibit the endometriotic lesion growth by inducing decidualization [21, 22] and also inhibiting implantation and angiogenesis of endometriotic lesions [23–25]. However, the exact mechanism of Dienogest on endometriotic lesion growth and development is still unclear and the controversial effect on endometrium thickness has been noted [26]. Nevertheless, recurrence of endometriosis after cessation of Esmya, Duphaston and Dienogest had never been studied.

In present study, we aimed to compare anti-endometriotic effects, reproductive side-effects, therapeutic mechanism and recurrence of Esmya, Duphaston and Dienogest in an experimental endometriosis mouse model.

## Methods

### Chemicals and reagents

17-β-estradiol was purchased from Sigma Chemical Co. (St. Louis, USA). Ketamine, xylazine, and acepromazine were purchased from Alfasan (Netherlands, Holland); Rabbit monoclonal antibody for Ki67 was purchased from Cell signaling technology (D3B5, Danvers, USA). In situ apoptosis detection kit was purchased from Merck Millipore (USA). Reagents for RT-PCR and quantitative PCR were purchased from TaKaRa (TaKaRa Bio, Shiga, Japan). Estradiol and progesterone ELISA kits were obtained from Cayman Chemical, USA. Prostaglandin E2 ELISA kit was purchased from Abcam (Abcam, England).

### Animals and endometriosis model

Six to seven weeks female C57BL/6 mice were housed in pathogen-free animal rooms with fixed cycle of 12 h’ light and 12 h’ dark. Standard laboratory chows and clean water were provided and all mice were allowed to acclimatize at least 1 week prior to experiments. A mixture of ketamine at 100 mg/kg, xylazine at 10 mg/kg and acepromazine at 3 mg/kg was injected intraperitoneally to anesthetize the mice before invasive surgery. Based on different goals of experiments in this study, two different mouse endometriosis models were established as below. Animals’ estrous cycle were synchronized by transferring urine-soaked male bedding every 5 days for both IP and SC endometriosis mouse model. [27]. All of the animal experiments were approved from the Animal Experimentation Ethics Committee, The Chinese University of Hong Kong.

### Intraperitoneal (IP) endometriosis mouse model for therapeutic safety and mechanistic study

The IP endometriosis model was established with minor modifications as described before [27, 28]. Briefly, mice were randomly divided into two groups, either donors or recipients. In day 0, uterus was dissected out from each donor mouse after sacrificed and washed in PBS for 3 times. Endometrial tissues were prepared from each uterus horn by a 2 mm biopsy punch. After endometrial tissues were prepared, recipient mouse was anesthetized and a 0.5 to 1 cm incision was made on the midline of mouse abdomen wall and peritoneum. Colon was pulled out carefully and kept hydration with sterile PBS. Three pieces of endometrial tissues were sutured on the vessels of mesentery with 6–0 surgical thread in each recipient mouse. Colon were put back to the peritoneal cavity gently; abdomen wall and skin were closed by 5–0 surgical thread individually. Mice were then placed on a warm pad until fully recovered from anesthesia after the surgery.

### Subcutaneous (SC) endometriosis mouse model for recurrence study

We established another mouse endometriosis model by SC transplantation to monitor the dynamic change of lesion growth during and after the medical interventions. It has been reported that both of IP and SC model exhibits similar development of endometriosis in respect with the cyst-like growth, and glandular and epithelial structures of the lesions [27, 29]. The same strain C57BL/6 mice were used to establish SC endometriosis mouse model as described before [30, 31]. Briefly, uterus was dissected out from donor mice and endometrial tissues were prepared as IP model mentioned above. Under anesthesia, the abdominal skin was shaved and then a 3 mm skin incision was made on the middle line of abdomen of each recipient mouse and bilateral SC pockets were created carefully without damaging the abdomen wall. One piece of endometrial tissue was placed into each pocket and then the skin incision was closed with 5–0 surgical thread.

### Study medications

One week after the surgery, mice were randomly divided into four groups. Either vehicle (the mice were treated with double distill water), Ulipristal acetate (Esmya™, 1 mg/kg, po, PregLem, England), Dydrogesterone (Duphaston™, 5 mg/kg, po, Abbott, USA) or Progestin (Dienogest™, 0.3 mg/kg, po, Bayer, Germany) were administered orally every day. The suggested oral daily dose for human from manufacturer instruction of Esmya, Duphaston and Dienogest were 5 mg, 20-30 mg and 2 mg, respectively. The dosage of each medication for the mice was based on FDA approved clinical dose for human and then converted to animal dose according to the guidelines of FDA calculator based on the surface area of humans and experimental animals [32]. All the medications were dissolved into appropriate volume of double distill water in 100ul.

For IP model, the medication was lasted for 28 days. For SC model, medication lasted for 21 days. The endometriotic lesion growth was determined by measuring longest length and perpendicular width of the lesions every 3 days in SC model and at the end of intervention in IP model using caliper as described before [31]. Then all the mice were sacrificed and lesions were removed and washed in sterilize PBS, then weighted on balance. For all lesions from each mouse, one lesion was fixed with 10% formalin (Sigma) and embedded in paraffin wax for histological analysis; the other lesions were either immersed in RNA later solution (Ambion™) for qPCR analysis or snap-frozen in liquid nitrogen for protein analysis.

Uterus size was estimated by measuring and averaging the longest diameter with caliper in the middle of each uterus horn. Ovaries was considered as ellipsoid and the ovary size was measured and calculated according to formula [33]: 1/6 π × length × width × thickness (mm^3^). Uterus was dissected and weighed before separated into 3 pieces and stored in the same way as lesions. Ovaries were dissected and then either fixed in 10% formalin for histological analysis or stored in RNA later solution for qPCR analysis.

### Hematoxylin and eosin staining

After embedding, 4um serial paraffin sections of lesions, uterus and ovary were prepared on coated slides. Hematoxylin and eosin staining will be carried out in every 10th section to confirm the microscopic structures in the lesions, uterus and ovary [34]. Surface area of the sections under the microscope were measured.

### Immunohistochemistry and TUNEL assay

Proliferation of the lesion was evaluated by immunohistochemical staining using rabbit anti mouse Ki-67 antibody (D3B5, Cell signaling technology). After de-paraffin and re-hydration, antigens were retrieved with sodium citrate buffer in microwave for 20 min. The sections were incubated with 1% BSA in PBS at room temperature for 1 h, follow by incubating with Ki-67 antibody in 4 °C for 15 h. After incubation with the primary antibody, sections were incubated with secondary antibody Donkey anti-Rabbit IgG-HRP (sc2313, Santa Cruz) for 1 h at room temperature and then with the colour was developed using DAB kit (k3468, Dako). Finally, the sections were counterstained with hematoxylin and dehydrated in serial concentration ethanol and mounted with coverslips. All the sections were examined under microscope. The cells with strong or moderated brown staining in nuclear were counted.

Apoptotic cells in the lesion were examined using an in situ apoptosis detection kit (ApopTAG®, S7100, Millipore, USA) according to the manufacturer’s instruction.

### Quantitative PCR analysis

Total RNA in the lesions was extracted by QIAGEN RNeasy Mini Kit (QIAGEN, Germany) according to the manufacturer’s protocol. Concentration and quality of RNA were measured by Nanodrop spectrophotometry (Wilmington, USA) and reverse-transcribed with TaKaRa RT-PCR kit (TaKaRa, 6110A, Japan) in ProFlex PCR System (Thermo Fisher Scientific, USA). Quantitative qPCR was carried out with TaKaRa SYBR Green quantitative qPCR kit (SYBR® Premix Ex Taq, TaKaRa) in 7900HT Fast Quantitative PCR system. Forty nanograms of cDNA was added to the reaction system. Primers used in this study are listed in Additional file 1: Table S1. Mouse β-actin gene was used as housekeeping gene. Data were analyzed using relative expression level and comparison was performed between different groups after normalized to the β-actin expression. The markers had been shown to play important role in the pathogenesis of endometriosis were chosen to screen the therapeutic actions [31]. Mmp2 and Plau play key role in breaking down the endometrial stroma and extracellular basement membrane [35, 36]. Integrins αV β3 (Itgav β3) is an adhesion molecule that functions in both cell–cell and cell–substratum adhesion [37]. Vascular endothelial growth factor (Vegf) and hypoxia inducible factor-1α (Hif1α) are key factors in angiogenesis [38]. Both Vegf and Hif1α expression were increased in endometriotic lesions [39]. NF-kB p105 and MAPK1 play a critical role in the regulation of cell proliferation and apoptosis [40, 41]. Pro-caspase 3 (pro-Cas3) is a key factor in caspase related apoptosis. Proliferating cell nuclear antigen (Pcna) is a protein associated with cell proliferation [42].

### Uterine gland count and endometrium thickness index

After stained by hematoxylin and eosin and examined under microscope, 6 serial uterine sections were scanned with Leica microscope in 100× magnification. For uterine gland count, all the glands in endometrium layer were counted and averaged for statistical analysis. Endometrium thickness index (ETI) was evaluated as the method described before with some modifications [43]. Briefly, the area of endometrium (A_e_) and uterus cavity (A_uc_) was measured using Image J [44]. ETI was then calculated using the formula $ETI=\sqrt{\frac{{\mathrm{A}}_{\mathrm{e}}}{\uppi}}-\sqrt{\frac{A_{uc}}{\pi .}}$

### Follicle count

After stained by hematoxylin and eosin and examined under microscope, 3 serial ovarian sections were scanned with Leica microscope in 40× magnification. Follicle classification was based on morphological criteria: primary follicles were defined as follicles which contain oocyte surrounded by single layer of granulosa cells; secondary follicles were defined as follicles which contain oocyte with 2 or more layers of granulosa cells, and antral follicles were defined as follicles contain oocyte which surrounded by several layers of granulosa cells and contain a visible antrum [45, 46]. The follicle count of each classification in each sample was measured and compared.

### Enzyme-linked immunosorbent assay

Serum and peritoneal fluid were collected for enzyme-linked immunosorbent assays. Whole blood was collected by 1 ml syringe with 25G needle from mouse heart, and then was centrifuged at 4 °C with 1000×g for 10 min. Serum was collected after centrifuge and stored in -80 °C. Peritoneal fluid was collected as method described before with some modifications [47]. Mice were sacrificed by overdose of anesthetic; skin was opened to expose the abdomen. 3 ml sterilize PBS and 2 ml air were injected into peritoneum using a 5 ml syringe and 21G needle. Then the peritoneal salvage was removed and centrifuged at 4 °C, 1000×g for 10 min, the supernatant was kept in -80 °C prior to analysis. Estradiol (E2) and progesterone (P4) levels in serum and prostaglandin E2 (PGE2) concentration in peritoneal fluid were measured by ELISA kits (Abcam, England) according to manufacturer’s instruction.

### Statistical analysis

Statistical analysis was carried out with Software Graphpad Prism 5, the differences between the treatment groups and control group were evaluated by One Way ANOVA if data distribution normal or Kruskal–Wallis test if data distribution skewed followed by Dunnett’s multiple post-hoc comparisons. Data were expressed as mean ± SEM. *p* < 0.05 was considered as significant.

## Results

### Ectopic endometriotic lesions growth

**Fig. 1:**
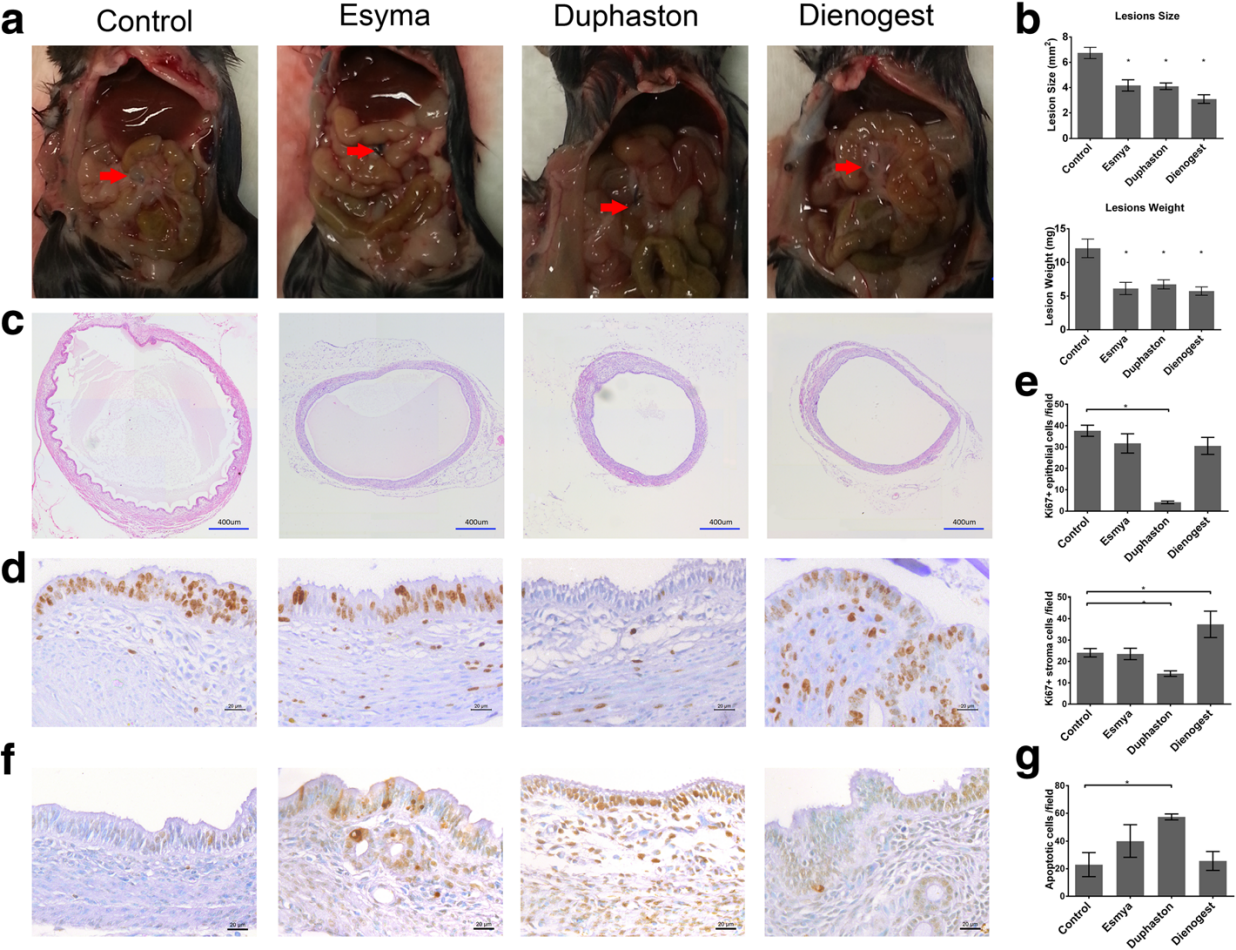
Esmya, Duphaston and Dienogest suppress the growth and development of endometriotic lesions in mice. **a** Lesion size was measured by caliper at the end of intervention. Representative in situ pictures of the endometriotic lesions in vehicle, Esmya, Duphaston and Dienogest group are shown. Red arrows indicate the locations of the lesions. **b** Endometriotic lesion size and weight. Data are shown as mean ± SEM, *n* = 9 for each group, *: *p* < 0.05 when compared to the control group. **c** Histological change of lesions after medication. This panels show the representative cross-sections of the endometriotic lesion as endometrial cyst-like structure. *Scar bar*: 400um. **d** Proliferation of the endometriotic lesions. Representative pictures of immunohistochemical staining of Ki67 in endometriotic-like lesions in different groups after treatment. *Scar bar*: 200um. **e** Comparison of Ki67 positively stained epithelial and stroma cells in the lesions. **f** Representative pictures of TUNEL staining in endometriotic-like lesions in different groups after treatment. *Scar bar*: 200um. **g** Comparison of TUNEL positively cells in the lesions. Data are shown as mean ± SEM, *n* = 3 for each group, *: *p* < 0.05 when compared to the control group

After IP transplantation, endometriotic lesions developed in the mesentery (Fig. 1a). Compared to vehicle control (size: 6.744±0.444 mm^2^; weight: 12.1±1.383 mg, *n* = 9), lesion size and weight were significantly decreased after treatment with Esmya (size: 4.171± 0.442 mm^2^, *p* < 0.0001; weight: 6.133±0.907 mg, *n* = 9, *p* = 0.0007), Duphaston (size: 4.104±0.262 mm^2^, *p* < 0.0001; weight: 6.747±0.679 mg, n = 9, *p* = 0.0012) and Dienogest (size: 3.092±0.340 mm^2^, *p* < 0.0001; weight: 5.744±0.621 mg, *n* = 9, *p* = 0.001) (Fig. 1b). The cyst-like endometriotic lesion with endometrial glandular and stroma structures were underdeveloped in the treatment groups (Fig. 1c). Cell proliferation in both epithelial and stromal cells of the endometriotic tissues was significantly decreased in Duphaston group when compared with control group (Fig. 1d and e, epithelial: 4.117±0.581 cells/field vs 37.606±2.604 cells/field, *n* = 9, *p* < 0.0001; stromal: 14.294±1.305 cells/field vs 23.258±1.783 cells/field, *n* = 9, *p* = 0.0095). However, cell proliferation in stromal cells were significantly increased in Dienogest group (37.333±6.179 cells/field vs 23.258±1.783 cells/field, *n* = 9, *p* = 0.0202). Apoptotic cells in the endometriotic lesions were significantly increased in Duphaston group. (Fig. 1f and g, 57.333±2.186 cells/field vs 23±8.756 cells/field, *n* = 9, *p* = 0.0461).

### Therapeutic actions

**Fig. 2:**
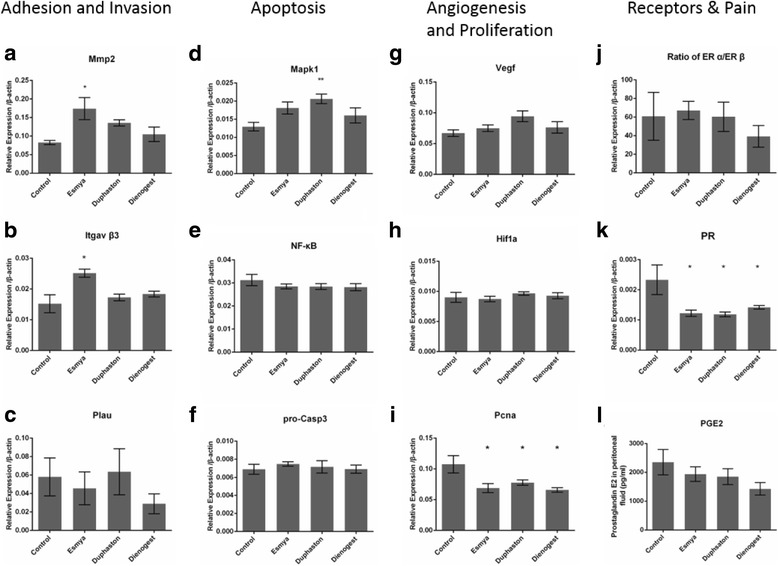
Comparisons of anti-endometriosis mechanism of Esmya, Duphaston and Dienogest. Relative mRNA expression of **a**-**c**, adhesion (Mmp2 and Itgav β3) and invasion (Plau) marker genes, **d**-**f**, apoptosis-related genes (Mapk1, NF-kB p105 and pro-Caspase 3), **g**-**i**, angiogenesis and proliferation mrakers (Vegf, Hif1α and Pcna) and **j**-**k**, hormone receptors (ERα/ERβ ratio and PR) in lesions; and ELISA quantitation of **l**, Prostaglandin E2 (PGE2) concentrations in peritoneal fluid. Data are shown as mean ± SEM, n = 9 for each group, *: *p* < 0.05 when compared to the control group

Only Esmya significantly increased the mRNA expression of adhesion markers Mmp2 (0.174±0.030 vs 0.083±0.006, *n* = 9, *p* = 0.0066) and Itgav β3 (0.025±0.001 vs 0.015±0.003, *n* = 9, *p* = 0.001), but not invasion marker Plau (0.046±0.018 vs 0.058±0.021, *n* = 9, *p* = 0.941) (Fig. 2a-c). Only Duphaston significantly increased the mRNA expression of apoptotic marker Mapk1 (0.021±0.001 vs 0.013±0.001, *n* = 9, *p* = 0.0138) but not NF-κB p105 (0.014±0.0002 vs 0.013±0.0012, *n* = 9, *p* = 0.686) and pro-Casp3 (0.0071±0.0006 vs 0.0068±0.0006, n = 9, *p* = 0.968) (Fig. 2d-f,). All Esmya, Duphaston and Dienogest significantly decreased the mRNA expression of proliferation marker Pcna (0.069±0.007 vs 0.078±0.004 vs 0.066± 0.004 vs 0.108±0.014, *p* = 0.0079, *p* = 0.0479, *n* = 9, *p* = 0.0041; Esmya, Duphaston, Dienogest and control, respectively), but not angiogenesis markers Vegf (0.0745±0.006 vs 0.0942±0.011 vs 0.080±0.009 vs 0.0675±0.006, *n* = 9, *p* = 0.825, *p* = 0.051, *p* = 0.777; Esmya, Duphaston, Dienogest and control, respectively) and Hif1α (0.0087±0.0004 vs 0.0097±0.0003 vs 0.0093±0.0005 vs 0.0090±0.0008, *n* = 9, *p* = 0.967, *p* = 0.739, *p* = 0.970; Esmya, Duphaston, Dienogest and control, respectively) (Fig. 2g-i). Esmya, Duphaston and Dienogest also significantly decreased the mRNA expression of progesterone receptor PR (0.0012±0.0001 vs 0.0012±0.00008 vs 0.0014±0.00006 vs 0.0023±0.0005, *n* = 9, *p* = 0.0099, *p* = 0.0099, *p* = 0.0168; Esmya, Duphaston, Dienogest and control, respectively) but not estrogen receptor ERα/ERβ ratios (67.151±9.706 vs 60.342±15.704 vs 39.284±11.690 vs 60.861±25.804, n = 9, *p* = 0.983, *p* > 0.999, *p* = 0.668; Esmya, Duphaston, Dienogest and control, respectively) (Fig. 2j-k). Pain mediator PGE2 concentration in peritoneal fluid was not significantly decreased after any treatment (1937.169±250.804 pg/ml vs 1850.524±273.428 pg/ml vs 1476.434±250.292 pg/ml vs 2484.759±466.669 pg/ml, *n* = 9, *p* = 0.720, *p* = 0.597 *p* = 0.152; Esmya, Duphaston, Dienogest and control, respectively) (Fig. 2l).

### Safety profiles

**Fig. 3:**
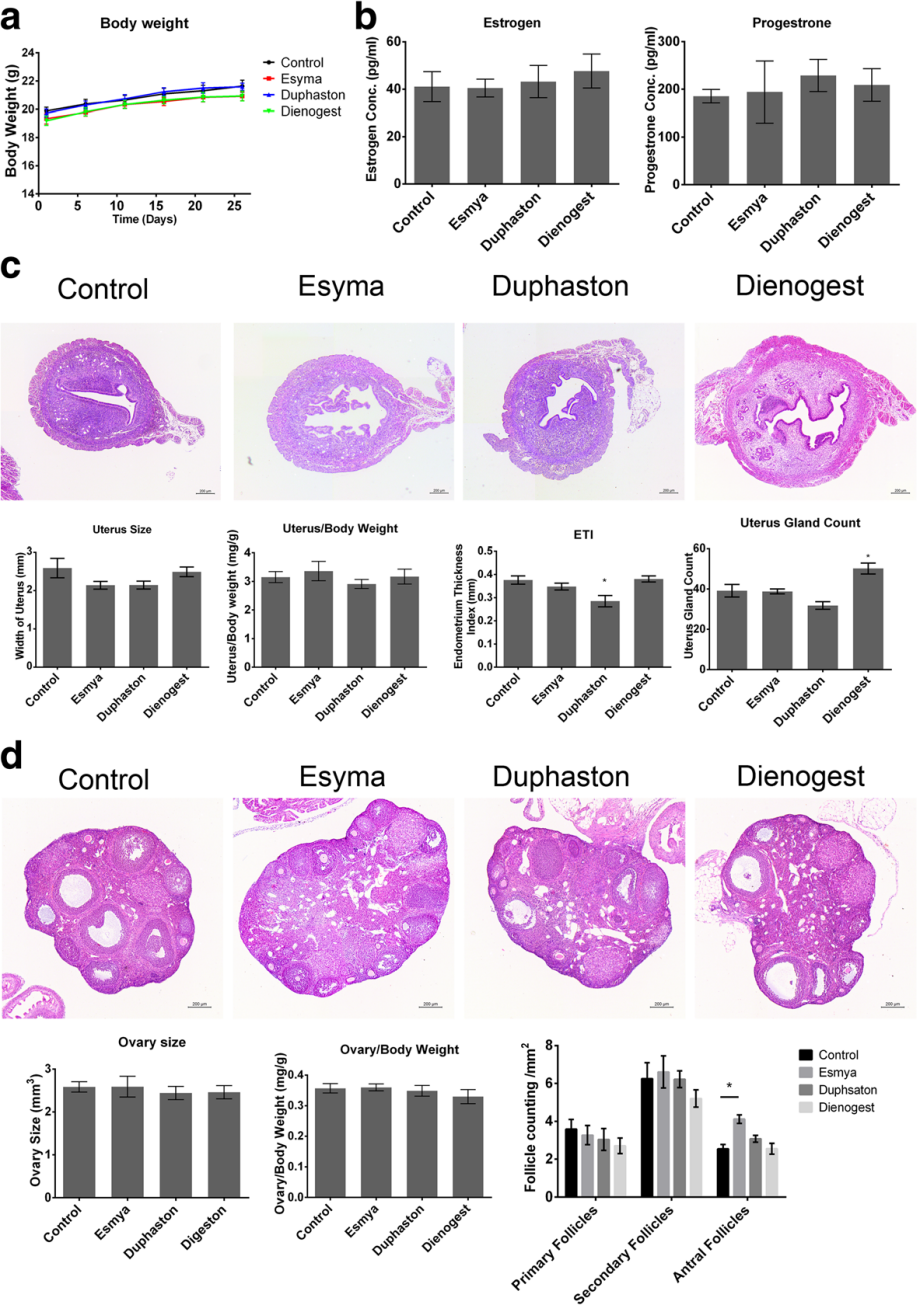
Safety profiles of Esmya, Duphaston and Dienogest. **a** Body weight changes during the study intervention. Data was recorded every 5 days from day 1 to day 25 after transplantation. **b** Serum estrogen and progesterone concentrations after treatment in the mice. **c** Upper panels show the representative cross section images of uterus, structure of endometrium gland, stroma and lumen cavity. *Scar bar*: 200um. Lower panels compare the uterus size, ratio of uterus and body weight, endometrium thickness index (ETI) and uterus gland count from left to right. **d** Upper panels show the histological change of ovary after treatment. *Scar bar*: 200um. Lower panels compared the ovary size, ratio of ovary and body weight and the follicle count from left to right. Data are shown as mean ± SEM. *: *p* < 0.05 when compared to the control group

All the animals were well-tolerated with the treatment and showed no significant change in body weight (detailed mean±sem and *p* value was provided in Additional file 1: Table S2) (Fig. 3a). None of the animals exhibited any behavior or signs of stress or toxic reaction throughout the study. Serum estrogen (40.484±3.771 pg/ml vs 43.201±6.825 pg/ml vs 47.674±7.219 pg/ml vs 41.055±6.335 pg/ml, *n* = 9, *p* = 0.999, *p* = 0.990, *p* = 0.792; Esmya, Duphaston, Dienogest and control, respectively) and progesterone (194.489±65.011 pg/ml vs 228.899±33.706 pg/ml vs 209.084±34.224 pg/ml vs 185.480±14.228 pg/ml, *n* = 9, *p* = 0.997, *p* = 0.779, *p* = 0.958; Esmya, Duphaston, Dienogest and control, respectively) levels were not significantly different amongst groups after treatment (Fig. 3b). There is no significant difference in uterus size (2.140±0.102 mm vs 2.143±0.103 mm vs 2.490±0.125 mm vs 2.590±0.254 mm, *n* = 9, *p* = 0.344, *p* = 0.350, *p* = 0.978; Esmya, Duphaston, Dienogest and control, respectively) and weight (3.361±0.341 mg/g vs 2.908±0.159 mg/g vs 3.170±0.260 mg/g vs 3.148±0.193 mg/g, *n* = 9, *p* = 0.929, *p* = 0.902, *p* > 0.999; Esmya, Duphaston, Dienogest and control, respectively), but histological examination showed significant reduced ETI in Duphaston group (0.285±0.024 mm vs 0.376±0.018 mm, *n* = 9, *p* = 0.0149) and significant increased endometrial gland counts in Dienogest group (48.478±2.379 vs 34.637±3.743, *n* = 9, p = 0.0149) (Fig. 3c). Similarly, there was no significant change in ovary size (2.591±0.242 mm^3^ vs 2.444±0.153 mm^3^ vs 2.463±0.154 mm^3^ vs 2.587±0.120 mm^3^, n = 9, *p* = 0.987, *p* = 0.907, p = 0.907; Esmya, Duphaston, Dienogest and control, respectively) and weight (0.360±0.015 mg/g vs 0.349±0.018 mg/g vs 0.330±0.023 mg/g vs 0.357±0.015 mg/g, *n* = 9, *p* = 0.999, *p* = 0.986, *p* = 669; Esmya, Duphaston, Dienogest and control, respectively) but the numbers of antral follicle were significantly increased in Esmya group (0.285±0.024 mm vs 0.376±0.017 mm, n = 9, *p* = 0.0143) (Fig. 3d).

### Withdrawn recurrence

**Fig. 4:**
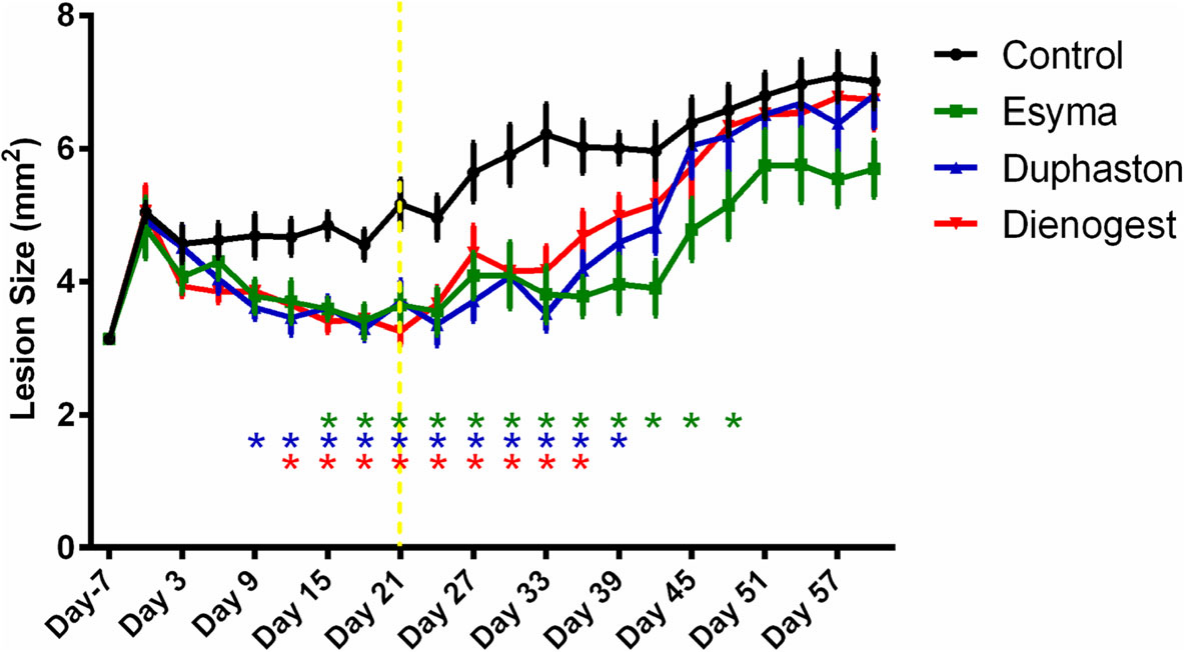
Dynamic change of endometriotic lesion size during and after treatment of Esmya, Duphaston and Dienogest. The endometriotic lesions were transplanted subcutaneously in day − 7. Intervention was started at day 0 and withdrawn at day 21 (yellow dot line). Data were shown as mean ± SEM, *n* = 10 for each group, *: *p* < 0.05 when compared to the control group in the corresponding days and groups

After SC transplantation, Duphaston significantly decreased the lesion size from day 9 (3.609±0.176 mm^2^, *n* = 10, *p* = 0.0418) of treatment, then Dienogest from day 12 (3.397±0.157 mm^2^, *n* = 10, *p* = 0.0024) and Esmya from day 15 (3.585±0.160 mm^2^, *n* = 10, *p* = 0.0223) when compared with control group (4.689±0.328 mm^2^ or 4.843±0.199 mm^2^ or 4.844±0.200 mm^2^, day 9, day 12, day 15 respectively) (Fig. 4). When treatment was withdrawn in day 21, lesion size of Dienogest group remained significantly smaller only until day 36 (4.686±0.377 mm^2^ vs 6.028±0.394 mm^2^, *n* = 10, *p* = 0.0168), 14 days after withdrawal. Lesion size of Duphaston and Esmya groups remained significantly smaller until day 39 (Duphaston vs control: 4.588±0.324 mm^2^ vs 6.001±0.233 mm^2^, *n* = 10, *p* = 0.0255) and day 48 (Esmya vs control: 4.962±0.556 mm^2^ vs 6.577±0.379 mm^2^, *n* = 10, *p* = 0.0352), 18 days and 27 days after withdrawal, respectively.

## Discussion

This is the first study to compare anti-endometriosis, therapeutic mechanism, safety profile and withdrawn recurrence of Esmya, Duphaston and Dienogest in an experimental endometriosis model in mice. The results showed that Esmya, Duphaston and Dienogest can effectively limit the endometriotic lesion growth and development (Table 1). The therapeutic effects are mainly through inhibition of progesterone receptor and cell proliferation and activation of apoptosis mechanisms. In particularly, Duphaston significantly inhibited the proliferation of both epithelial cells and stroma cells and activated apoptosis in the endometriotic lesions. Although wide safety margin, Duphaston induced endometrial atrophy, Dienogest enhanced endometrial glandular hyperplasia, Esmya increased adhesion of the lesion and antral follicle accumulation, and recurrence shortly after Dienogest and Duphaston withdrawn.

**Table 1:**
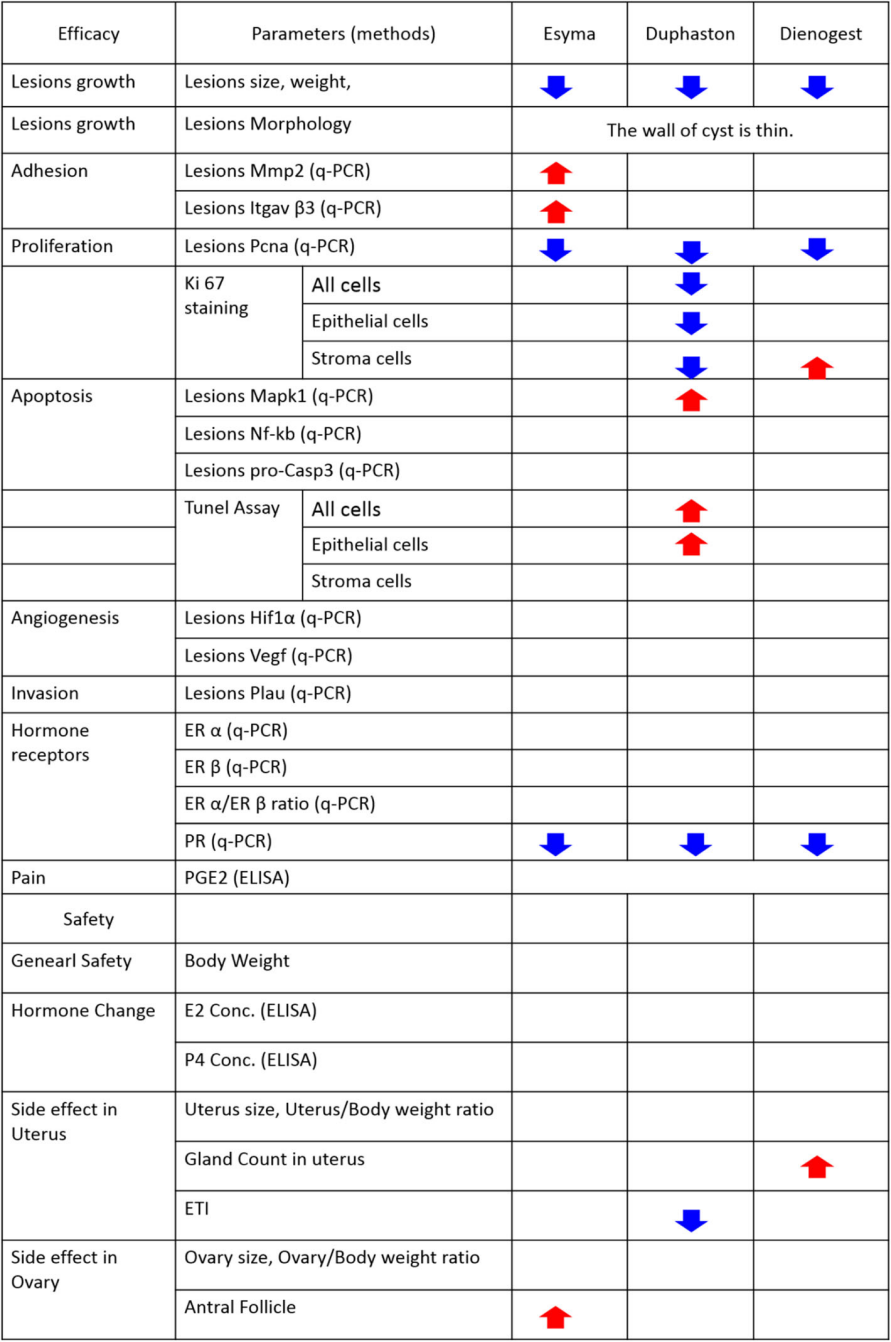
Summary of significant change after treatment of Esmya, Duphaston and Dienogest

Red arrows indicate significant increase and blue arrows indicate significant decrease when compared to the vehicle group

Esmya as a new generation SPRM has been used to treat uterine fibroids and as an emergency contraceptive [48, 49], but it is not yet used for treatment of endometriosis. The major active component of Esmya is ulipristal acetate. The detail mechanism of ulipristal acetate on endometriosis of human is still not clear. Ulipristal acetate had been tested as another brand of medication, ellaOne, on a rat endometriosis model before [50]. According to Huniadi’s study, 0.1 mg ulipristal acetate daily for 8 weeks significantly reduced the endometriotic lesion about to 55%. The potential therapeutic effects of ulipristal acetate on increasing apoptosis and decreasing proliferation were also confirmed in this study although its safety was not studied. Compared to our study, the inhibitory effect was similar in terms of the decreased lesion size and proliferation. Although our dose was similar in terms of human equivalent dose, our intervention time was shorter (4 weeks vs 8 weeks). It has been shown that ulipristal acetate increased Bax/Bcl-2 ratio and cytochrome c expression [50] which activated capase-3 and increased apoptosis in the endometriotic lesions [51]. In our study, we did not study cytochrome c and Bax/Bcl-2 but neither apoptotic markers Mapk1, NF-κB p105 or pro-caspase3 expression were significantly increased in the lesions. The limitation of mRNA expression level is that only the pro-caspase-3 rather than activated form caspase-3 can be evaluated. The expression level of pro-caspase-3 on mRNA level can provide one of evidence for the caspase-related apoptosis in endometriotic lesions, however, the definitive regulation of apoptosis of endometriotic lesions should be carried out by protein expression, such as immunohistochemistry or Western blot analysis. Unlike caspase related apoptosis, Mapks involved in the pathophysiological process of endometrium decidualisation [52] and upregulated NF-κB p105 [53, 54]. When compared to Esmya, Duphaston has similar anti-endometriosis efficacy in reducing the lesion size to about 61%. But only Duphaston significantly reduced proliferating cells in both epithelial and stroma cells and significantly increased apoptotic cells and Mapk1 expression in the endometriotic lesions. Although cells proliferation markers Pcna and Ki67 specifically correlated with each other [55, 56], Ki67 positive cells in endometriotic lesions were significantly decreased in Duphaston group only. In contrast to the previous publication [22], Dienogest significantly increased Ki67 cells in the stromal cells but significantly decreased Pcna expression in endometriotic lesions. Compared with Esmya and Duphaston, Dienogest has the strongest anti-endometriosis efficacy in terms of the reduced lesion size up to 61%. Its anti-proliferative and anti-apoptosis effects on the endometriotic lesions were not as good as Esmya and Duphaston, suggesting other more important underlying mechanism may be involved in Dienogest, further study is needed, thus warranting further future studies.

Some common side effects of progestin include, but not limit to hot flushes, irregular uterine bleeding, headache and weight gain [21, 57]. SPRMs have similar side effects, like hot flushes, headache and nausea, but SPRMs significantly reduce abnormal uterine bleeding in uterine fibroid [58]. In our study, no significant change on body weight gain and behavioural changes were observed, indicating wide safety margin amongst Esmya, Duphaston, Dienogest. Increased weight gain after long-term administration of progestin for 6 months or above was demonstrated in other study [59–61], but for short-term treatment as in our study, no significant change in body weight was observed in mice. In uterus, Esmya, Duphaston and Dienogest showed different adverse effects probably due to different effects of SPRMs and progestin [62]. Ulipristal acetate can control irregular bleeding by inducing amenorrhea [58, 63, 64] while Dienogest may cause irregular uterine bleeding unless cyclic administration or combine estradiol valerate treatment [65, 66]. Due to the progesterone antagonist effect of SPRMs, it is well known that ulipristal acetate can induce the progesterone receptor modulator associated endometrial changes (PEAC) [67], but no significant change in endometrial morphology was observed in Esmya group in our study. It may be due to short-term administration of Esmya in our intervention protocol and the high metabolic and rapid cyclic changes of endometrium in mice [68]. Consistent with previous studies, by inducing withdrawal bleeding Duphaston could suppress endometrial hyperplasia which may lead to lower endometrium thickness [69–71] observed in our study. Similar to Duphaston, Dienogest may also reduce endometrium thickness [72–74], but further study showed that endometrium thickness change under the treatment of Dienogest are mainly dependent on the duration of treatment [26]. In our short-term study, there was no significant endometrium thickness change, but in contrast endometrial glandular hyperplasia was observed in Dienogest group. In ovary, both Duphaston and Dienogest are as effective progestins that can be used as oral contraceptive with different ovulation inhibitory effects [75, 76]. The ovulation inhibitory effect induced by Dienogest could be reverse rapidly by stopping treatment [74]. Interestingly, Duphaston showed different anti-ovulation effect on different species [75]. Whilst Duphaston induces delay effect on ovulation in rats, it only slightly inhibits ovulation in rabbits but has no effect on ovulation in monkeys [75]. No significant change on ovary and follicles development was observed in our Duphaston and Dienogest groups, but antral follicles was significantly increased in Esmya group. The accumulation of antral follicles may be related to delay in follicular rupture induced by ulipristal acetate [49, 77]. On the other hand, recurrence of endometriosis is very common. The reported recurrence rate was high, estimated as 21.5% at 2 years and 40–50% at 5 years [78]. All the pharmacological treatments used are able to control the symptoms but not effectively treating the condition [79]. In our study, the endometriosis growth was still inhibited after withdrawal of Esmya, but not after Duphaston and Dienogest. This may be related to the prolong anti-ovulation effect of Esmya while the anti-endometriosis effects of Duphaston and Dienogest through inhibiting decidualisation and ovulation is rather transient. The detailed mechanism about Duphaston and Dienogest on recurrence of endometriosis is still unknown. Taken together, endometrium side-effects and withdrawn recurrence of Duphaston and Dienogest should be warranted.

Although Dienogest can be used as contraceptive medication, Dienogest showed no significant effect on endometrium in short term treatment in our study and ovulation inhibition could be reversed by stopping intervention in another study [74]. Based on the characteristics of Esmya on delaying ovulation and the suppressive effect of Duphaston on endometrium growth, they maybe not suitable for endometriosis patients who are considering conception. Because of the anti-proliferation effect on endometrium and suppressive effect on endometriotic lesions, Duphaston could be a better option for endometriosis patient during perimenopause, while Esmya is suitable for endometriosis patient who cannot tolerate progesterone effect due to the heavy irregular uterine bleeding. Even though Esmya have been used for contraceptive and it shows suppressive effect on endometriotic lesion growth, the detailed mechanism of anovulation and accumulation of antral follicle after treatment with Esmya is still unknown. Further studies should be carried out to understand the dynamic changes and detailed mechanism of anovulation effect for its further use in endometriosis. For Duphaston, its potential progesterone effect on ovulation is still unknown, so further studies should also be carried out to investigate its detail effect on ovary.

There are three major limitations in our present study. In order to compare the efficacy and safety, all three drugs were tested on an experimental model of endometriosis in mice. The therapeutic effect was tested in an animal which have no menstruation and never develop endometriosis spontaneously. The endometriosis was induced by suturing endometrium tissues on the vessels of mesentery or placing endometrium tissues into SC pocket rather than inducing by IP injection. When compared to the possible pathogenesis process of endometriosis, induced endometriosis by suturing endometrial tissues may cause extra inflammatory reaction on the endometriotic lesions. However, compared to the IP injection model, suturing or placing endometrial tissues to induce endometriosis has a better control on the variation and monitoring of the lesion size and weight. Furthermore, all these medications were tested in only single dosage and only for 4 weeks. The dose was used according to the FDA approved dose for human use and the duration already covered at least 6 estruses cycle of the mice which equivalent to almost half year in human. The purpose of present study was to compare progestins and selective progesterone receptor modulator as new indication for endometriosis, and the results of the study already showed positive therapeutic effects in clinical dose. Hence higher dose and longer treatment duration may not be necessary. The markers were selected to screen the therapeutic actions, not for detailed mechanistic study. Finally, the samples were collected at the end of intervention, therapeutic effect and side effect were compared in only a single time point which is not sufficient to monitor the dynamic change of reproductive organs or the hormone change during intervention.

## Conclusion

All Esmya, Duphaston and Dienogest can suppress endometriotic growth on mouse model and Dienogest has the best suppressive effect. Esmya has significant ovulation suppressive effect and Duphaston may suppress endometrium growth while no significant side effect of Dienogest on ovary or endometrium was found. Detail endometriotic lesion suppressive effect and side effect on ovary or endometrium are still not clear and further studies need to be carried out.

## Additional file

Additional file 1: Primer sequences of qPCR. (DOCX 18 kb)
