# Supplementary material for: Efficacy, safety and recurrence of new progestins and selective progesterone receptor modulator for the treatment of endometriosis: a comparison study in mice
Source: Reprod Biol Endocrinol. 2018 Apr 3;16:32. doi: 10.1186/s12958-018-0347-9 (PMC5883298; doi:10.1186/s12958-018-0347-9)
Supplement: Supplementary file 1 — Primer sequences of qPCR. (DOCX 18 kb) [file 12958_2018_347_MOESM1_ESM.docx]

Table 1, Primers used for screening therapeutic actions

| Markers | Genes | Primers | Product size |
| --- | --- | --- | --- |
| Adhesion | Mmp2 | 5-ACGGGCCCTATCATCTTCAT-3 | 137 |
|  |  | 5-CACAAAAAGAAGCCACCCTCT-3 |  |
|  | Itgav β3 | 5-ACCACTAACATCACCTGGGG-3 | 203 |
|  |  | 5-TCTTCTTGAGGTGGTCGGAC-3 |  |
| Invasion | Plau | 5-CTAGAGCCCTGGAGCCTCTT-3 | 192 |
|  |  | 5-TGGGATGGTCTATGCTGTCA-3 |  |
| Apoptosis | Mapk1 | 5-TTTGCATAGGGAGGTCCAAG-3 | 150 |
|  |  | 5-GGTGCCATCATCAACATCTG-3 |  |
|  | Nfkb p105 | 5-GCACAGACGGTGTCTAGCAA-3 | 130 |
|  |  | 5-CGGAGGGACAGCAGTAACA-3 |  |
|  | Pro-Casp3 | 5-TGGTGATGAAGGGGTCATTTATG-3 | 105 |
|  |  | 5-TTCGGCTTTCCAGTCAGACTC-3 |  |
| Angiogenesis | Vegf | 5-GCCAGACAGGGTTGCCATAC-3 | 108 |
|  |  | 5-GGAGTGGGATGGATGATGTCAG-3 |  |
|  | Hif1α | 5-ACCTTCATCGGAAACTCCAAAG-3 | 187 |
|  |  | 5-ACTGTTAGGCTCAGGTGAACT-3 |  |
| Proliferation | Pcna | 5-TTGCACGTATATGCCGAGACC-3 | 183 |
|  |  | 5-GGTGAACAGGCTCATTCATCTCT-3 |  |
| Receptors | Estrogen receptor α | 5-CCTCCCGCCTTCTACAGGT-3 | 128 |
|  |  | 5-CACACGGCACAGTAGCGAG-3 |  |
|  | Estrogen receptor β | 5-CTGTGATGAACTACAGTGTTCCC-3 | 80 |
|  |  | 5-CACATTTGGGCTTGCAGTCTG-3 |  |
|  | Progesterone receptor | 5-CTCCGGGACCGAACAGAGT-3 | 122 |
|  |  | 5-ACAACAACCCTTTGGTAGCAG-3 |  |
| House keeping | β-actin | 5-GGGACCTGACAGACTACCTCAT-3 | 185 |
|  |  | 5-GTCAGGCAGCTCATAGCTCTTC-3 |  |

Table 2, Summary of statistical analysis results of body weight change during intervention.

|  |  | Control | Esmya | Duphaston | Dienogest |
| --- | --- | --- | --- | --- | --- |
| Day 1 | Mean | 19.878 | 19.344 | 19.733 | 19.189 |
|  | SEM | 0.257 | 0.379 | 0.283 | 0.333 |
|  | P value vs control | | 0.501 | 0.977 | 0.302 |
|  |  |  |  |  |  |
| Day 6 | Mean | 20.367 | 19.778 | 20.300 | 19.822 |
|  | SEM | 0.325 | 0.254 | 0.275 | 0.285 |
|  | P value vs control | | 0.344 | 0.997 | 0.406 |
|  |  |  |  |  |  |
| Day 11 | Mean | 20.644 | 20.333 | 20.733 | 20.333 |
|  | SEM | 0.335 | 0.262 | 0.303 | 0.291 |
|  | P value vs control | | 0.804 | 0.993 | 0.804 |
|  |  |  |  |  |  |
| Day 16 | Mean | 21.089 | 20.544 | 21.233 | 20.633 |
|  | SEM | 0.398 | 0.290 | 0.283 | 0.255 |
|  | P value vs control | | 0.472 | 0.976 | 0.606 |
|  |  |  |  |  |  |
| Day 21 | Mean | 21.311 | 20.844 | 21.500 | 20.856 |
|  | SEM | 0.387 | 0.334 | 0.373 | 0.332 |
|  | P value vs control | | 0.682 | 0.966 | 0.697 |
|  |  |  |  |  |  |
| Day 26 | Mean | 21.633 | 20.922 | 21.589 | 20.933 |
|  | SEM | 0.426 | 0.325 | 0.254 | 0.353 |
|  | P value vs control | | 0.344 | 0.999 | 0.356 |
